# Pre-and-post-operative aversion among men whose partners had caesarean delivery in a patriarchal setting

**DOI:** 10.4314/gmj.v55i4.9

**Published:** 2021-12

**Authors:** Abiodun S Adeniran, Olumuyiwa O Ogunlaja, Idowu P Ogunlaja, Shukurat B Okesina, Adegboyega A Fawole, Kikelomo T Adesina, Abiodun P Aboyeji

**Affiliations:** 1 Obstetrics & Gynaecology Department, University of Ilorin/ University of Ilorin Teaching Hospital, Ilorin, Nigeria; 2 Department of Obstetrics & Gynaecology, Bowen University, Iwo / Bowen University Teaching Hospital, Ogbomoso, Nigeria; 3 Obstetrics & Gynaecology Department, General Hospital, Ilorin, Nigeria; 4 Obstetrics & Gynaecology Department, State Specialist Hospital, Ilorin, Nigeria

**Keywords:** Caesarean delivery, aversion, perception, male partner, patriarchal setting

## Abstract

**Objectives:**

The study evaluated pre and post-operative perception and aversion to caesarean delivery (CD) among men whose partners underwent the procedure.

**Design:**

A multicentre cross-sectional study.

**Setting:**

Two tertiary and two secondary health facilities.

**Participants:**

Men whose partners underwent CD at the study sites.

**Methods:**

Participants were recruited by purposive sampling, data collection was through interaction via an interviewer-administered questionnaire first immediately the decision for CD was made and thereafter on the third postoperative day. Men whose partners had vaginal delivery were excluded from the study and data management was with SPSS version 21.0 while p<0.05 was significant.

**Results:**

Awareness about CD was 84.0% mainly through the healthcare workers (42.1%) and the female partner (34.1%); 88.0% of participants recommended CD for medically-indicated reasons. The greatest influence on consent was the male partner (48.8%). The major pre-operative concerns were limitation of family size (34.7%) and fear of repeat CD (34.0%). Pre-operative perceptions of CD included being expensive (60.7%), fear of the procedure (48.0%), fear of complications (45.3%) and longer hospital stay (44.0%). Aversion to CD was 30.0% pre and 5.3% post-operation; predictors of aversion were history of previous surgery among male or female partner and awareness about CD. However, there were reductions in negative perception and aversion post-operation.

**Conclusion:**

The high negative perception and aversion to CD among male partners were reduced post-operation. Healthcare workers should address the concerns and negative perceptions about CD and prioritize patient-friendly experiences during surgical operations.

**Funding:**

Funding was by the researchers; no grant or external support was received for the study.

## Introduction

In low resource countries especially Sub-Saharan Africa, despite the awareness about caesarean delivery (CD), many women are averse to it due to desire for vaginal delivery, fear of surgery or anaesthetic complications, concerns about repeat CD and possible death.^1^–^3^ Other concerns include increased duration of hospital stay, delay in resuming household chores and limitation of family size.^4^,^5^

In addition, some women experienced poor reception at home or criticism from their partners after undergoing CD.^6^ Generally, studies on aversion to CD have focused mainly on women; a study reported aversion rates of 71.7% before and 20.9% after the procedure among women who had CD.^3^

Male partner involvement in reproductive health refers to the various ways in which men relate to reproductive health problems, programmes and reproductive behaviour.^7^

It also includes all forms of assistance and support provided by men to improve their partners' and children's health during pregnancy, labour and delivery.^8^ The gender role model assign roles in households and communities based on sex such that in patriarchy, men hold the position of power and decision-making in all issues involving members of the household. On the other hand, women are limited in self decision-making on healthrelated issues including access to healthcare, financial power to pay for treatment and the capacity to give consent for CD thereby prolonging the waiting time and increasing life threatening complications.^9^,^10^ In a study on aversion to CD among women who required the procedure, the male partner was reported to be the overriding influence in giving consent in 26.8% of cases.^3^ Therefore, although CD reduces maternal and perinatal morbidity/ mortality when indicated; access to and acceptance by women to undergo CD requires the active involvement of men.

While research has evaluated aversion to CD among women in patriarchal settings; the responses of the male partners remain understudied although men are an important determinant of the response of women to the procedure. Therefore, as we continue to encourage male participation in reproductive health, their dominant role in decision-making should be explored to make their participation beneficial. The study aimed at evaluating the perception and aversion to CD among male partners of women who had CD by conducting a pre-and postoperative evaluation among these men.

## Methods

The study was a prospective cross-sectional study conducted at two secondary and two tertiary health facilities in Nigeria. These facilities were chosen because they were the main public referral hospitals offering CD in the study area. Participants were the male partners of women who had emergency CD at the study sites during the study period. The inclusion criteria were being the male partner of the woman who underwent the CD and an informed consent to participate in the study. Other male relations and partners of women who had vaginal deliveries were excluded from the study.

The sample size was calculated using the formula for cross-sectional study.^11^ Using a prevalence of 7% for aversion among male partners,^12^ a confidence level of 95%, a degree of accuracy of 0.05% and attrition rate of 20%, the minimum sample size for the study was 120. However, 150 participants were recruited to increase the power of the study.

The sampling technique employed was purposive sampling in which all consenting male partners of women for CD at the study sites were recruited until the sample size is completed. The male partners of all women who had CD were informed and counselled about the study after the decision for the procedure has been made and an informed consent was obtained. A pre-operative interviewer-administered questionnaire was administered to each participant to determine the perception and aversion to CD before the procedure while a post-operative evaluation was conducted on the third day after the surgery.

The third day was chosen to provide opportunity for the respondents to have had enough experience for post-operative evaluation. The study questionnaire included demographic factors, perception about CD before the surgery, events surrounding the surgery including the response when informed of the need for CD, issues about consent, and influences in accepting the procedure as well as the post-operative perception of CD. The data obtained from the study was analyzed using the Statistical Package for Social Sciences (SPSS software version 21.0) with level of significance set as p value <0.05.

Ethical approval was obtained from the ethical review committee of the University of Ilorin Teaching Hospital, Ilorin, Nigeria (Approval number ERC PAN/2016/03/1504) before commencement of the study and an informed consent was obtained from all participants in the study.

## Results

There were 150 participants, the mean age was 37.5±6years (range 22 to 53 years), 107(71.3%) had tertiary education, 126(84.0%) were aware of CD and the common sources of information were healthcare workers in 53(42.1%) and the female partner in 43(34.1%). Also, 132(88.0%) of participants opined that CD should be performed only when medically indicated; 98(65.3%) of the female partners have had previous surgery out of which 95(96.9%) were previous CD as shown in Table 1.

**Table 1:** Biosocial characteristics of the male partners

Parameter	Frequency n=150 (%)
**Age group (years)**	
**20–29**	10 (6.7)
**30–39**	87 (58.0)
**40–49**	45 (30.0)
**50–59**	8 (5.3)
**Mean age ± SD**	37.5±6 (range 22–53)
**Occupation**	
**Civil servant**	83 (55.3)
**Artisan**	9 (6.0)
**Business/ trader**	58 (38.7)
**Level of education**	
**None**	4 (2.7)
**Primary**	8 (5.3)
**Secondary**	31 (20.7)
**Tertiary**	107 (71.3)
**Wife had surgery before**	
**No**	52 (34.7)
**Yes**	98 (65.3)
**Type of surgery the wife had (n=98)**	
**Caesarean delivery**	95 (96.9)
**Myomectomy**	3 (3.1)
**Awareness about CD**	
**Yes**	126 (84.0)
**No**	24 (16.0)
**Source of information about CD (n=126)**	
**Books**	8 (6.4)
**Radio**	12 (9.5)
**Relatives**	16 (12.7)
**Television**	17 (13.5)
**Friends**	21 (16.7)
**Internet**	29 (23.0)
**My wife**	43 (34.1)
**Health workers**	53 (42.1)
**Opinion about CD**	
**Should be the normal mode of delivery**	6 (4.0)
**Should be on request by the couple**	12 (8.0)
**Should be performed when medically indicated**	132 (88.0)

* More than one answer allowed

From Table 2, 115(76.7%) of participants imagined that the partner might have CD, 82(71.3%) of them went for prayers while 12(10.4%) discussed with the health worker. The greatest influence in giving consent for the CD was the husband in 73(48.8%) while 8(5.3%) did not agree that the partner should undergo the CD.

**Table 2:** Response, consent taking and other perioperative events among participants

Parameters	Frequency n=150 (%)
**Imagined wife might deliver via CD**	
**Yes**	115 (76.7)
**No**	35 (23.3)
**Action taken after the thought (n=115)**	
**Told my mother**	2 (1.7)
**Believed it was a spiritual attack**	5 (4.4)
**Shoved it aside**	6 (5.2)
**Told my partner**	8 (7.0)
**Told the health worker**	12 (10.4)
**Went for prayers**	82 (71.3)
**Greatest influence in giving consent**	
**In-laws**	3 (2.0)
**Religious leader**	3 (2.0)
**Male partner's relatives**	5 (3.3)
**Husband's friends**	5 (3.3)
**Wife/ female partner**	11(7.3)
**Health worker**	50 (33.3)
**Husband/male partner**	73 (48.8)
**Male partner's response to need for CD**	
**Did not agree that partner should have CD**	8 (5.3)
**Agreed that partner should have CD**	142 (94.7)
**Pre-op concern of male partners** *	
**Comment of others**	3 (2.0)
**May be unable to pay the bill**	20 (13.3)
**Baby might die**	22 (14.7)
**Wife/ partner might die**	45 (30.0)
**Wife/ partner may require CD for next delivery**	51 (34.0)
**May limit number of children wife/ partner can** **have**	52 (43.7)
**Who should give consent for CD?**	
**The husband's parents**	2 (1.3)
**The woman**	6 (4.0)
**The couple**	63 (42.0)
**The husband**	79 (52.7)
**Male partner's awareness about indication for** **CD**	
**Aware**	127 (84.7)
**Unaware**	23 (15.3)
**Response when informed that partner needed CD**	
**Broke down and wept**	12 (8.0)
**Refused initially**	12 (8.0)
**Asked for time to inform my religious leader**	17 (11.3)
**Agreed with the decision**	109 (72.7)
**Possible response if partner needs CD in future**	
**Disapprove of the procedure**	8 (5.3)
**Willingly accept**	98 (65.4)
**I don't know**	44 (29.3)
**Advise to a friend whose partner needs CD**	
**Encourage husband to disapprove of the procedure**	4 (2.7)
**Encourage him to give consent**	123 (82.0)
**I don't know**	23 (15.3)

* Multiple answers allowed

The major pre-operative concerns of the men were that CD will limit the number of children the partner could have in 52(34.7%) while 51(34.0%) believe the partner will require CD in future deliveries.

While 79(52.7%) opined that the male partner should give consent for CD, 63(42.0%) want it to be a couple decision and 6(4.0) want the wife to give consent. When informed about the need for CD, 109(72.7%) agreed without delay, 98(65.4%) will willingly consent while 123(82.0%) will encourage a friend to consent to wife's CD.

From Table 3, the pre-op perceptions of the male partners include that it was expensive by 91(60.7%), 72(48.0%) perceived it is to be feared, 68(45.3%) perceived it has many complications while 66(44.0%) perceived CD as a cause of longer hospital stay.

**Table 3:** Comparative analysis of perception to CD before and after the procedure among the male partners

Perception about CD	Before CD n=150 (%)	After CD n=150 (%)	χ^2^	P value
**Expensive**	91(60.7)	74(49.3)	54.623	<0.001
**Like a death sentence**	13(8.7)	5(3.3)	54.509	<0.001
**Fear of anaesthesia**	25(16.7)	8(5.3)	20.704	<0.001
**It could harm my baby**	11(7.3)	14(9.3)	28.674	<0.001
**It could harm my wife**	33(22.0)	28(18.7)	35.872	<0.001
**Has many complications**	68(45.3)	44(29.3)	47.112	<0.001
**Possible blood transfusion**	28(18.7)	14(9.3)	15.057	<0.001
**A dangerous procedure**	40(26.7)	18(12.0)	40.496	<0.001
**It is for those without faith**	4(2.7)	3(2.0)	0.084	0.722
**Fear of surgical procedure**	72(48.0)	29(19.3)	17.401	<0.001
**It means future CD**	22(14.7)	31(20.7)	67.869	<0.001
**It reduces number of children**	46(30.7)	51(34.0)	37.398	<0.001
**Causes longer hospital stay**	66(44.0)	54(36.0)	14.836	<0.001

CD: Caesarean delivery

However, there was a significant positive improvement in all perceptions post CD except among those with the perception that CD is for people without faith (p=0.722).

From table 4, aversion to CD was reported among 45(30.0%) participants' pre and 8(5.3%) post CD.

**Table 4:** Assessment of aversion and its determinants pre and post caesarean delivery among the male partners

Parameter	Pre-operative aversion Total participants= 150				Post-operative aversion Total participants=150			
	Yes (n=45)	No (n=105)	χ^2^	P value	Yes (n=8)	No (n=142)	χ^2^	P value
**Occupation**								
**Artisan**	4(8.9)	4(4.8)	1.437	0.488	1(12.5)	8(5.6)	1.082	0.582
Self-employed	15 (33.3)	43(40.9)			2(25.0)	56(39.5)		
**Civil servant**	26(57.8)	57(54.3)			5(62.5)	78(54.9)		
**Level of education**								
**None**	2(4.4)	2 (1.9)	6.602	0.158	0(0.0)	4(2.8)	0.832	0.934
**Primary**	0(0.0)	8(7.6)			0(0.0)	8(5.6)		
**Secondary**	9(20.0)	22(20.9)			2(25.0)	29(20.4)		
**Tertiary**	34(75.6)	73(69.5)			6(75.0)	101(71.1)		
**Previous surgery (man)**								
**Yes**	8(17.8)	5(4.8)	6.742	0.009	0(0.0)	13(9.2)	0.802	0.371
**No**	37(82.2)	100(95.2)			8(100.0)	129(90.8)		
**Previous surgery (woman)**								
**Yes**	17(37.8)	81(77.1)	21.552	0.001	6(75.0)	92(74.8)	0.349	0.555
**No**	28(62.2)	24(22.9)			2(25.0)	50(35.2)		
**Awareness about CD**								
**Yes**	35(77.8)	91(86.7)	1.852	0.174	4(50.0)	122(85.9)	7.269	0.007
**No**	10(22.2)	14(13.3)			4(50.0)	20(14.1)		
**Consented to the CD**								
**Yes**	43(95.6)	99(94.3)	0.100	0.751	3(37.5)	139(97.9)	54.699	0.000
**No**	2(4.4)	6(5.7)			5(62.5)	3(2.1)		
**Blood transfusion (woman)**								
**Yes**	14(31.1)	24(22.9)	1.135	0.287	2(25.0)	36(25.3)	0.001	0.982
**No**	31(68.9)	81(77.1)			6(75.0)	106(74.7)		

The significant predictors of aversion pre-operation were history of previous surgery among male partner (p=0.009), previous surgery in the female partner (p=0.001) while awareness about CD (p=0.007) and consent of the male partner to the CD (p=0.001) were significant post operation.

## Discussion

Although awareness about CD among the men in the study was lower (84.0%) than 93.3% among pregnant women in a similar study,^3^ the men's major source of information about CD were the health workers and the female partners.

This suggests an effective communication among health workers and antenatal clinic attendees as well as a stepdown of the information from the women to their partners. Therefore, health workers should continue to prioritize health talks and other avenues to educate pregnant women on health-related issues as its impact is extended to their partners.

In recent times, efforts have focused on reducing the rising global CD rate especially by preventing unnecessary CD. Therefore, the opinion of 88.0% of men in this study that CD should be medically indicated is a positive development. A report from Louisiana indicated that the probability for potentially unnecessary CD was 17/100 for primary and 43/100 for repeat CS.^13^ Thus, efforts should be intensified to prioritize and encourage medically indicated CD. It was observed that 76.7% of men in this study at some time in the antenatal period imagined that the female partner could have a CD; 10.4% of them discussed this with the health worker while 71.3% approached the religious leader for support. This underscores the need for improved engagement of the male partners by health care workers during antenatal clinic visits to create an enabling environment for counselling. This further supports the call by pregnant women for antenatal classes for the male partners^14^ to provide additional opportunity for education and counselling on the concerns of these men.

Obtaining informed consent for CD is often a challenge in patriarchal cultures; in this study, 52.7% of participants opined that it is the right of the male partner to give consent, 42.0% favoured a joint decision by the couple while 4.0% agreed that the woman can give consent. Also, the major influence for accepting the procedure was the male partner in 48.8%, the health worker in 33.3% and the female partner in 7.3%. In a study from Southeast Nigeria, 90% of the participating pregnant women wanted the male partner to sign the consent, 6% favoured the woman while 4% supported that any of the couple can give consent.^15^ In another study among pregnant women in a patriarchal culture, 82% of pregnant women were willing to undergo CD if the male partners consent to it despite the woman's disapproval because men are the head of the family and the women believe that men want what is best for them and the baby.^15^ This underscores the psychology of patriarchal culture where the man is assumed to be right in all his decisions. However, although 5.3% of the male participants disagreed that the woman should have CD, the in-laws and relations overruled the decision and the women had the surgery. This highlights the common overriding influence of in-laws in decision making in patriarchal cultures where they are also believed to be affected by the outcome of such decisions.

Pre-operative concerns of the male partners included possible limitation of number of future children, fear of repeat CD, death of the woman or baby and possible inability to pay the hospital bill. In a similar study, the male partners were concerned about the severity of the labour pain and viewed the decision for CD as an opportunity to relieve the labour pain.^16^ In another report, 32.1% of male partners were concerned about possible death of the woman, 14.9% feared limitation in the number of future children while 10.3% were bothered by possible complications after the surgery.^17^ In another study, the male partners were anxious about the life and safety of the wife and child and considered the consequences of the woman's death on the family.^16^ The anxiety among the male partners was heightened by the duration of surgery; the men described the waiting period as being long and time appeared to be moving too slowly while the kept looking at the watch.^1^

The commonest perception of men about CD were that the procedure was expensive, dangerous, associated with complications and leads to prolonged duration of hospital admission. Generally, CD is expensive compared to vaginal delivery while families in low-income countries fail to capitalize on the cushion provided by health insurance in augmenting the cost. In a study among women who had CD, 54.6% paid out-of-pocket because they did not enrol for health insurance; thus, the amount they paid for CD was significantly higher than the contribution of those on health insurance.^18^ Another study showed that CD was associated with a mean duration of 71.7 hours compared to about 24 hours for most vaginal deliveries.^19^

In this study, aversion to CD among male partners was 30% pre and 5.3% post-operation while previous surgery by either the male partner or the woman and awareness about CD were statistically significant determinants of aversion. In a similar report on aversion among women undergoing CD, aversion was 71.7% before and 20.9% after the surgery.^3^ The reduction in aversion post-operation has been linked to the counselling and the outcome of the CD. Male partners were reported to be relieved on seeing both the woman and baby in good condition after CD with eventual support for CD as the right delivery option.^16^ In another report, the authors reported previous surgery to be associated with increased aversion for CD. Therefore, the authors recommended that medical personnel should prioritize patient safety as well as other interventions to make the surgical experience patient friendly as this influences the attitude to CD in the future.^3^

The role of religion and religious leaders in decision making on health-related issues continues to attract attention globally. In this study, religious leaders were the first contacted for advice by the men following the thought that the woman may deliver by CD in 71.3% of cases, the first to be called after decision for CD in 11.3% and the major influence of consent to the procedure in 2.0% of cases. It has been reported that religious leaders can play an important role in the health behaviours of their congregants by influencing decision making at individual, congregational and communal levels.^20^ Therefore, they can be partnered in health promotion, empowered with the correct information with which to facilitate favourable disposition and acceptance of CD for themselves, their congregants and the community.

The study recommends that healthcare workers should provide enabling environment to discuss concerns and perceptions of women and their partners about CD throughout pregnancy, labour and delivery. In addition, efforts should be made to scale up uptake of health insurance for women of reproductive age and their families to reduce the cost of maternity services. Health workers should intensify efforts to make surgeries safer and patient-friendly while religious leaders should be partnered to improve the perception and response to CD among the populace.

## Conclusion

The study concludes that negative perception and aversion to CD remains high in the pre-operative period among male partners of women who underwent CD although these improved in the assessment post-surgery.
